# From Stent to Septic shock: A Rare Case of Tumor Ingrowth Through an Uncovered Self-Expandable Metal Stent Causing Life-Threatening Cholangitis in Pancreatic Head Carcinoma

**DOI:** 10.7759/cureus.97234

**Published:** 2025-11-19

**Authors:** Faizan Sheraz

**Affiliations:** 1 Department of Internal Medicine, Hospital Corporation of America (HCA) / Sunrise Health Graduate Medical Education Consortium, Las Vegas, USA

**Keywords:** ascending cholangitis, diagnostic and therapeutic ercp, pancreatic-biliary cancer, self-expanding metal stents (sems), septic shock (ss)

## Abstract

We report a rare case of rapid tumor ingrowth causing complete occlusion of an uncovered self-expandable metal stent (SEMS) within two months of placement in a 65-year-old female patient with pancreatic head adenocarcinoma. The patient developed septic shock secondary to ascending cholangitis, requiring intensive care unit admission. Endoscopic retrograde cholangiopancreatography revealed complete stent occlusion due to tumor ingrowth. Successful salvage was achieved through coaxial placement of a covered SEMS through the occluded stent, with resolution of sepsis and restoration of biliary drainage. This case highlights the aggressive nature of pancreatic adenocarcinoma and the importance of vigilant monitoring for early stent failure, particularly in patients awaiting chemotherapy, where treatment delays can significantly impact survival outcomes.

## Introduction

Endoscopic biliary stenting remains a cornerstone in the palliative management of malignant biliary obstruction, particularly in pancreatic head cancer. Self-expandable metal stents (SEMSs) have revolutionized the approach to biliary drainage in unresectable pancreatic adenocarcinoma, offering superior patency compared to plastic stents. Uncovered SEMSs are traditionally favored for their lower migration rates and longer patency compared to plastic stents, with reported median patency times ranging from 6 to 12 months [1,2]. However, tumor ingrowth through the stent mesh represents a significant limitation, occurring in approximately 27.6% of patients and potentially leading to stent occlusion, recurrent biliary obstruction (RBO), and life-threatening cholangitis [3].

The clinical significance of stent-related complications extends beyond immediate patient morbidity. Recent multicenter data demonstrate that cholangitis episodes occur in approximately 20% of pancreatic cancer patients undergoing neoadjuvant therapy, with median overall survival reduced from 36 months to 26 months in affected patients [4]. Furthermore, chemotherapy interruptions caused by stent failure affect 16% of patients and are independently associated with reduced overall survival (OS) and progression-free survival (PFS) [4]. This underscores the critical importance of optimal stent selection and management strategies.

Tumor ingrowth represents a unique pathophysiologic process distinct from other causes of stent occlusion. Unlike tumor overgrowth at stent ends or sludge impaction, tumor ingrowth involves neoplastic tissue proliferation through the stent mesh, creating intraluminal narrowing and eventual complete occlusion [3]. This phenomenon is particularly problematic in pancreatic adenocarcinoma due to the aggressive nature of the malignancy and its propensity for rapid local progression. Recent studies have identified specific risk factors for tumor ingrowth, including the use of uncovered and laser-cut SEMS designs, sharp common bile duct (CBD) angulation, and high axial-force stent characteristics [3,5].

The rarity of this case lies not in the occurrence of tumor ingrowth per se, but in the rapid timeline of stent occlusion (two months post-placement) leading to septic shock, and the successful management using a coaxial covered SEMS placement technique. This case exemplifies the evolving understanding of stent selection criteria and the importance of individualized approaches based on tumor biology and patient anatomy. With emerging data on novel stent designs, including double-slim systems (DSSs), polytetrafluoroethylene-coated stents, and hybrid designs with strategic perforations, the landscape of biliary stenting continues to evolve [6,7].

## Case presentation

A 65-year-old woman with recently diagnosed pancreatic head adenocarcinoma presented with progressive jaundice, generalized weakness, and abdominal pain. Two months prior, the patient underwent placement of an uncovered SEMS in the CBD to reduce the risk of stent migration. Chemotherapy was scheduled to begin the following week.

**Figure 1:**
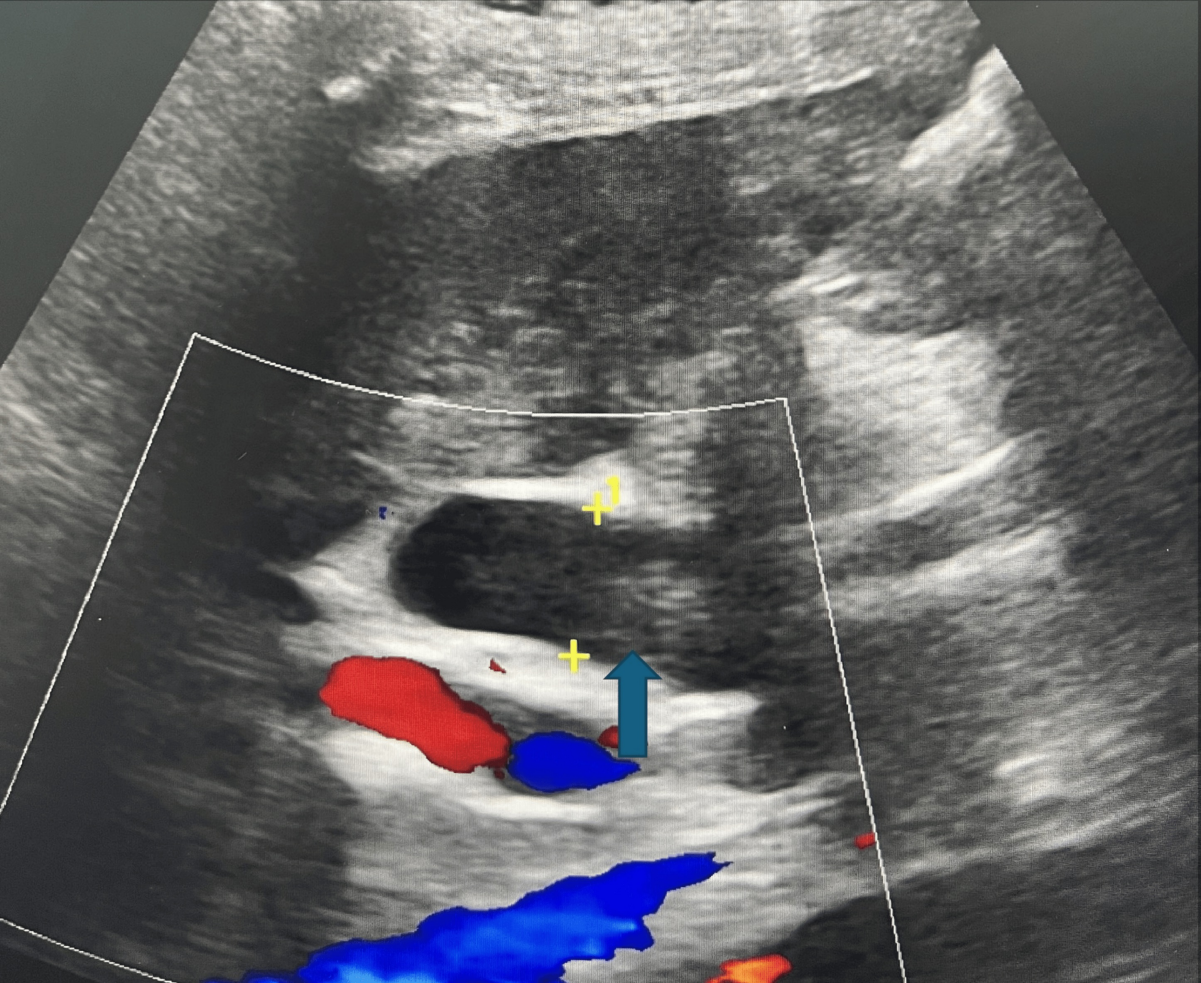
Ultrasonographic image showing a dilated common bile duct.

**Figure 2:**
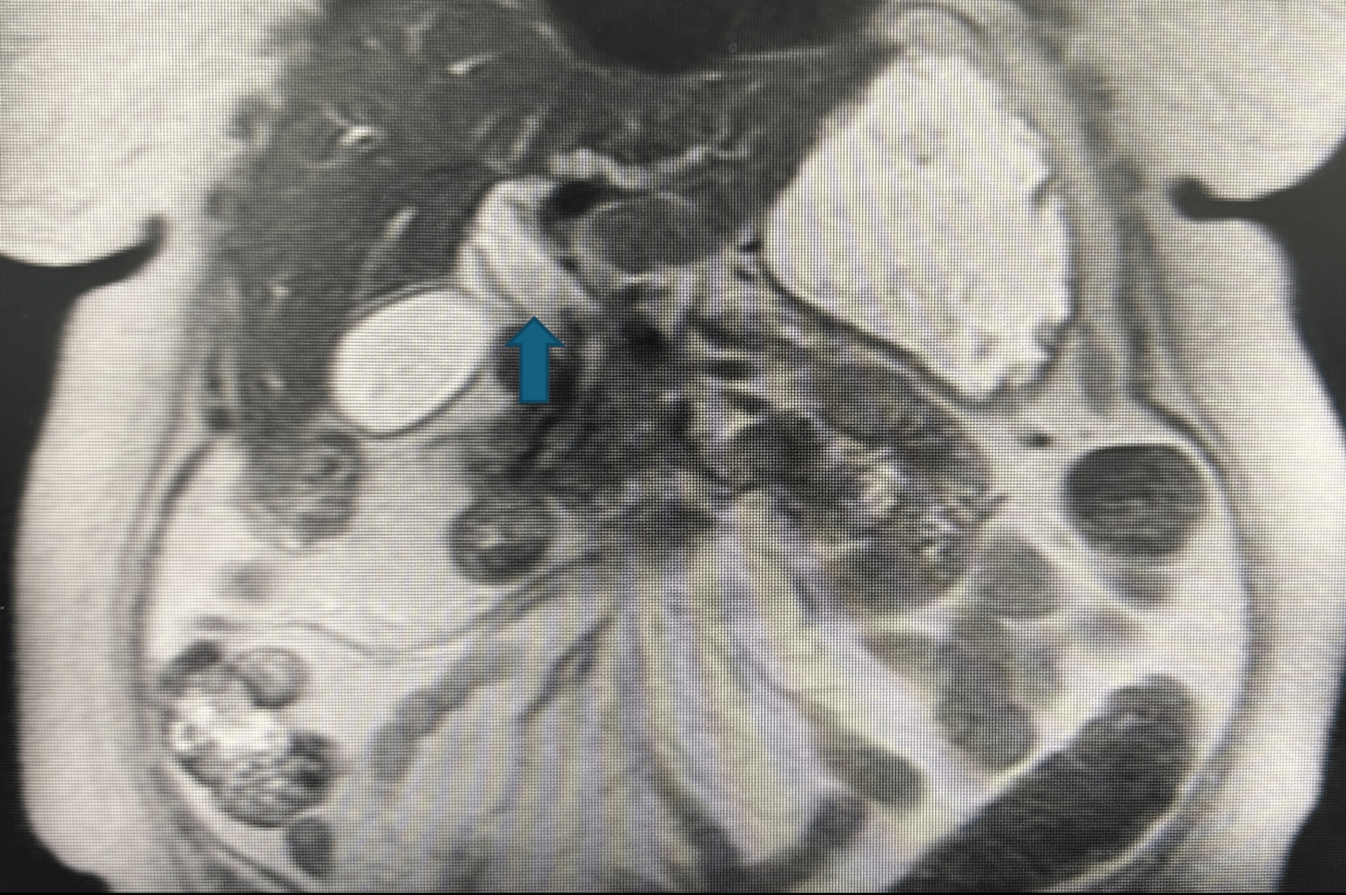
Magnetic resonance image showing dilated biliary ducts.

On admission, she was hypotensive with a blood pressure of 70/40 mmHg (normal reference range: 120/80), febrile with a temperature of 38.3 °C (normal reference range between 36.5°C and 37.2°C), and exhibited scleral icterus. Laboratory evaluation revealed a total bilirubin of 10 mg/dL (normal reference range 0.1-1.0 mg/dl). Liver function tests were markedly elevated. Abdominal ultrasound (Figure 1) demonstrated gallbladder sludge and a dilated CBD. Magnetic resonance imaging (MRI) of the abdomen (Figure 2) showed intra- and extrahepatic biliary dilation, a filling defect within the CBD, atrophy of the pancreatic body and tail, and pancreatic ductal dilation.

**Figure 3:**
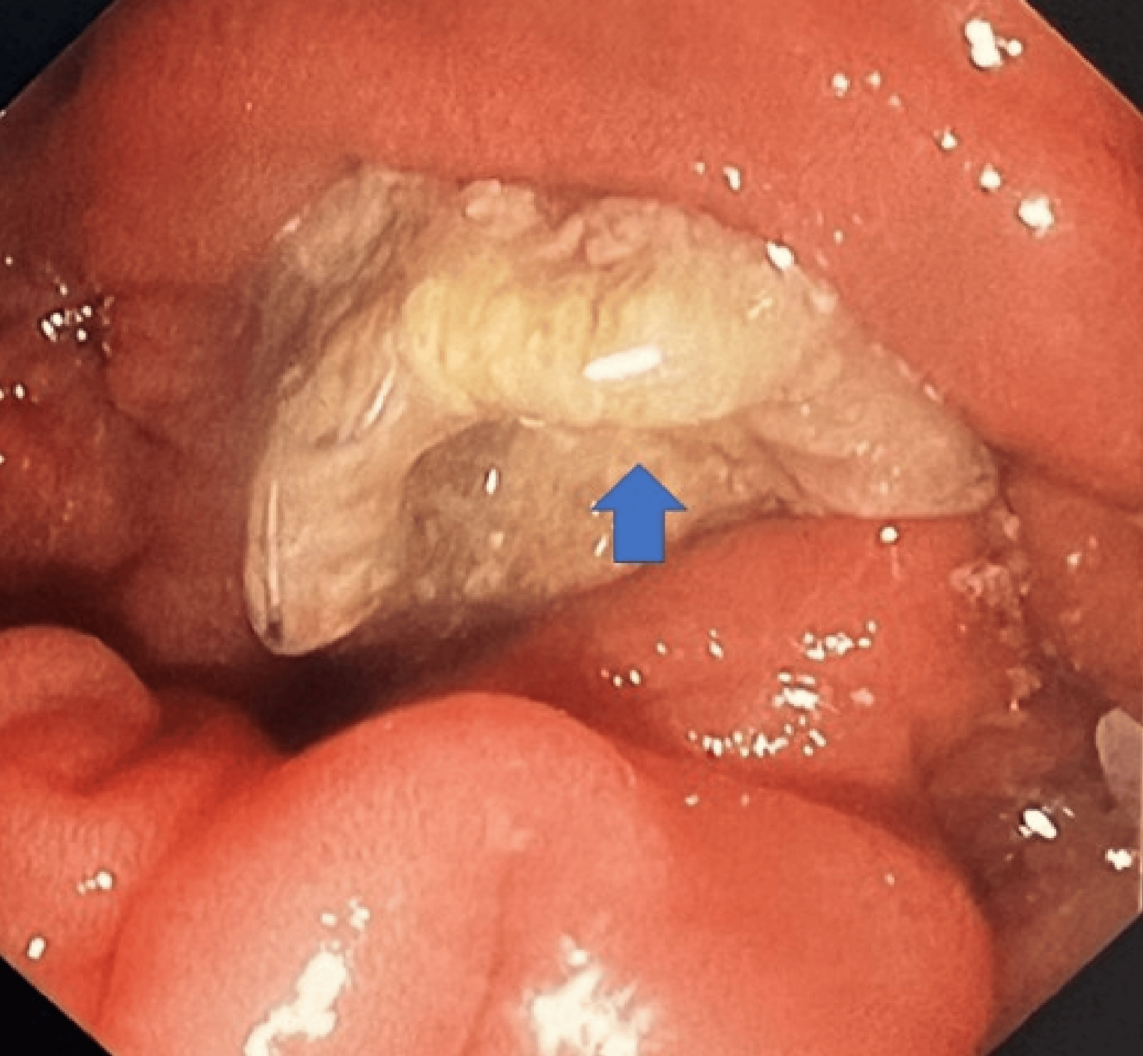
Endoscopic view showing growth of a tumor through an uncovered self-expanding metallic stent. Endoscopic appearance shows tumor ingrowth through the stent mesh causing luminal compromise.

**Figure 4:**
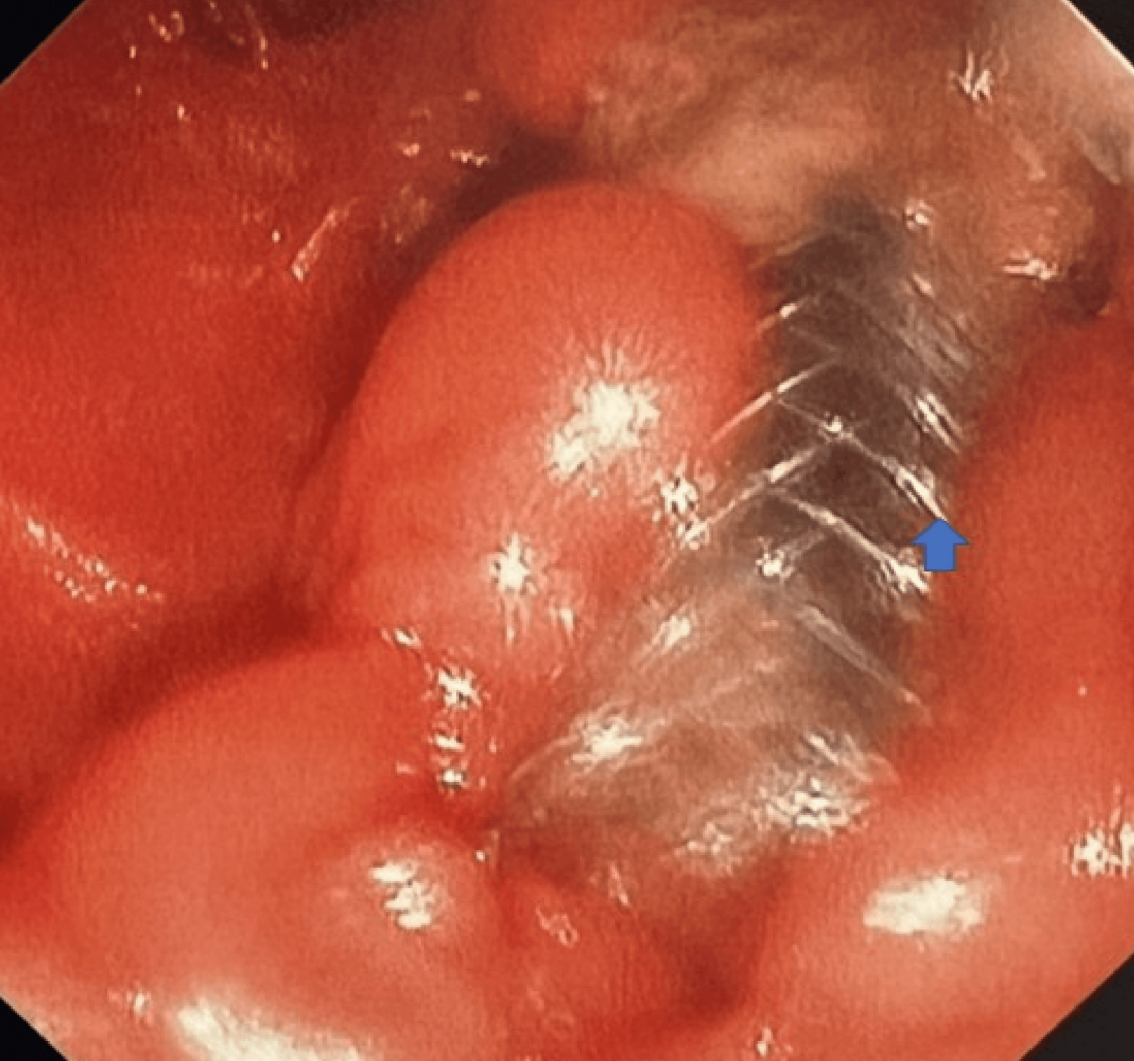
Endoscopic view showing successful placement of a covered self-expanding metallic stent. Endoscopic view demonstrates proper stent deployment with adequate luminal patency and intact stent covering.

She was admitted to the intensive care unit (ICU) with septic shock secondary to ascending cholangitis. Blood cultures were obtained and showed no growth. Endoscopic retrograde cholangiopancreatography revealed a visibly occluded uncovered metal stent at the major papilla (Figure 3). Tumor ingrowth was suspected as the cause of in-stent stenosis. Biliary sweeping yielded purulent drainage. A covered SEMS was placed coaxially through the previously deployed stent (Figure 4). Post-procedure, the patient's liver function tests improved, and her septic shock resolved with intravenous antibiotics.

## Discussion

This case illustrates a serious complication of uncovered SEMS in pancreatic cancer: in-stent stenosis due to tumor ingrowth occurring within an unusually short timeframe of two months. The rapid progression to septic shock underscores the aggressive nature of pancreatic adenocarcinoma and its propensity for early stent failure through tumor ingrowth mechanisms.

Pathophysiology and risk factors

Tumor ingrowth represents one of several distinct mechanisms leading to stent occlusion, alongside tumor overgrowth, sludge impaction, and migration [3]. The pathophysiology involves neoplastic tissue proliferation through the interstices of uncovered stent mesh, creating progressive luminal narrowing. Recent data from 162 patients identified independent risk factors for this complication, including the use of uncovered and laser-cut SEMS designs, which were significantly associated with tumor ingrowth compared to covered alternatives [3].

Our patient's case aligns with several identified risk factors. The use of an uncovered SEMS and the aggressive biology of pancreatic adenocarcinoma likely contributed to the rapid onset of tumor ingrowth. Additionally, anatomic factors such as sharp CBD angulation, which has been newly recognized as an independent predictor of shorter time to RBO, may have played a contributory role [5].

Comparison with contemporary literature

The timeline of stent occlusion in our patient (two months) is notably shorter than reported median patency times for uncovered SEMS, which typically range from 6 to 12 months in pancreatic cancer patients [1,2]. However, this aligns with emerging data showing significant variability in patency based on tumor biology and stent characteristics. Recent studies report RBO rates of 27.6% overall, with tumor ingrowth being a leading cause of failure in uncovered designs [3].

The clinical presentation with septic shock secondary to cholangitis mirrors findings from recent cohort studies demonstrating that cholangitis occurs in approximately 20% of pancreatic cancer patients, with a significant impact on survival outcomes [4]. The association between stent failure and chemotherapy interruption, affecting 16% of patients in recent series, emphasizes the oncologic implications beyond immediate morbidity [4].

Management strategies and outcomes

Our management approach using coaxial covered SEMS placement represents an established salvage strategy for tumor ingrowth. It was ensured that the guidewire passed through the lumen of the occluded stent and not through its mesh, which is key to a successful coaxial placement. This technique has demonstrated efficacy in multiple series, with recent data showing improved patency compared to repeat uncovered stent placement [8]. The choice of covered SEMS for the salvage procedure was based on its ability to resist tumor ingrowth while accepting the increased risk of migration and potential cholecystitis.

Recent comparative studies have refined our understanding of stent selection. DSSs have shown superior outcomes compared to conventional covered SEMS, with RBO rates of 30% versus 43% and median time to RBO of 378 days versus 195 days, respectively, [6]. Novel polytetrafluoroethylene-coated designs have demonstrated promising results with reduced tumor ingrowth rates while maintaining acceptable migration profiles [7].

Emerging technologies and future directions

The landscape of biliary stenting continues to evolve with innovative designs addressing the limitations of traditional SEMSs. Novel approaches include tapered-and-flared fully covered designs that optimize anchoring while preventing ingrowth, and hybrid stents with strategic perforations that balance patency with migration resistance [9,10]. Additionally, adjunctive therapies such as intraductal radiofrequency ablation before stent placement have shown promise in prolonging patency and improving survival metrics in randomized controlled trial data [11].

For patients with failed transpapillary drainage, endoscopic ultrasound-guided hepaticogastrostomy using a dedicated cautery-enhanced tubular SEMS has emerged as an alternative, with reported RBO rates as low as 5% in specialized centers [12].

Clinical implications

This case highlights several critical clinical considerations. First, the selection of stent type should consider individual risk factors, including tumor biology, anatomic features such as CBD angulation, and patient prognosis. Second, close monitoring of patients with uncovered SEMS is essential, particularly in aggressive malignancies like pancreatic adenocarcinoma. Third, prompt recognition and management of stent occlusion are crucial to prevent septic complications and minimize chemotherapy interruptions.

The successful outcome in our patient, with resolution of septic shock and restoration of biliary drainage, demonstrates the effectiveness of timely endoscopic intervention. However, the broader implications extend to optimal primary stent selection, with growing evidence supporting covered or novel hybrid designs in patients with aggressive tumors and anatomic risk factors.

Limitations and future research

While our case demonstrates successful management of tumor ingrowth, several questions remain unanswered. The optimal timing for prophylactic stent exchange, the role of adjunctive ablative therapies, and the comparative effectiveness of emerging stent technologies require further investigation through prospective randomized trials. Additionally, the development of predictive models incorporating tumor biology, anatomic factors, and stent characteristics could guide personalized stent selection strategies.

## Conclusions

In patients with pancreatic cancer, uncovered SEMSs may lead to early stent occlusion due to tumor ingrowth, resulting in biliary obstruction and cholangitis with significant oncologic implications. This case demonstrates that tumor ingrowth can occur within two months of stent placement in aggressive malignancies, leading to life-threatening complications. Covered SEMS placement through the occluded stent represents an effective salvage strategy, though primary prevention through optimal stent selection remains paramount.

The evolving understanding of risk factors for tumor ingrowth, including stent design characteristics and anatomic features, should inform clinical decision-making. As novel stent technologies and adjunctive therapies continue to emerge, a personalized approach to biliary drainage that considers tumor biology, patient anatomy, and individual risk factors will likely optimize outcomes. This case underscores the importance of vigilant monitoring, prompt intervention, and the need for continued innovation in biliary stenting techniques for pancreatic cancer patients.
